# Identification and description of three families with familial Alzheimer disease that segregate variants in the *SORL1* gene

**DOI:** 10.1186/s40478-017-0441-9

**Published:** 2017-06-09

**Authors:** Håkan Thonberg, Huei-Hsin Chiang, Lena Lilius, Charlotte Forsell, Anna-Karin Lindström, Charlotte Johansson, Jenny Björkström, Steinunn Thordardottir, Kristel Sleegers, Christine Van Broeckhoven, Annica Rönnbäck, Caroline Graff

**Affiliations:** 1 Department NVS, Center for Alzheimer Research, Division for Neurogeriatrics, Karolinska Institutet, Huddinge, Sweden; 2 Department of Geriatric Medicine, Genetics unit, Karolinska University Hospital, Stockholm, Sweden; 3 Neurodegenerative Brain Disease Group, VIB Center for Molecular Neurology, VIB, Antwerp, Belgium; 4 Laboratory of Neurogenetics, Institute Born-Bunge, University of Antwerp, Antwerp, Belgium

**Keywords:** Alzheimer disease, Whole-exome sequencing, SORL1, Inherited, Familial

## Abstract

Alzheimer disease (AD) is a progressive neurodegenerative disorder and the most common form of dementia. The majority of AD cases are sporadic, while up to 5% are families with an early onset AD (EOAD). Mutations in one of the three genes: amyloid beta precursor protein (*APP*), presenilin 1 (*PSEN1*) or presenilin 2 (*PSEN2*) can be disease causing. However, most EOAD families do not carry mutations in any of these three genes, and candidate genes, such as the sortilin-related receptor 1 (*SORL1)*, have been suggested to be potentially causative. To identify AD causative variants, we performed whole-exome sequencing on five individuals from a family with EOAD and a missense variant, p.Arg1303Cys (c.3907C > T) was identified in *SORL1* which segregated with disease and was further characterized with immunohistochemistry on two post mortem autopsy cases from the same family. In a targeted re-sequencing effort on independent index patients from 35 EOAD-families, a second *SORL1* variant, c.3050-2A > G, was found which segregated with the disease in 3 affected and was absent in one unaffected family member. The c.3050-2A > G variant is located two nucleotides upstream of exon 22 and was shown to cause exon 22 skipping, resulting in a deletion of amino acids Gly1017- Glu1074 of SORL1. Furthermore, a third *SORL1* variant, c.5195G > C, recently identified in a Swedish case control cohort included in the European Early-Onset Dementia (EU EOD) consortium study, was detected in two affected siblings in a third family with familial EOAD. The finding of three *SORL1*-variants that segregate with disease in three separate families with EOAD supports the involvement of SORL1 in AD pathology. The cause of these rare monogenic forms of EOAD has proven difficult to find and the use of exome and genome sequencing may be a successful route to target them.

**Electronic supplementary material:**

The online version of this article (doi:10.1186/s40478-017-0441-9) contains supplementary material, which is available to authorized users.

## Introduction

Alzheimer disease (AD) is the most common form of dementia and it is characterized by progressive neurodegeneration and deterioration of cognitive functions [4]. The neuropathological hallmarks of AD seen in brain are extracellular amyloid β (Aβ)-plaques and intracellular neurofibrillary tangles which primarily consist of hyperphosphorylated tau [3, 4, 19]. The disease usually has an age of onset after 65 years, but there are patients with an early onset, before the age of 65 with an the estimated prevalence of dementia of 35 per 100.000 individuals [13]. A fraction of familial early onset forms of AD can be explained by mutations in one of three genes: amyloid beta precursor protein (*APP*), presenilin 1 (*PSEN1*) and presenilin 2 (*PSEN2*) [5, 7]. The involvement of these three genes in AD was discovered more than two decades ago and these mutations only explain a minority of the families with inherited early onset Alzheimer disease (EOAD). The frequency with which mutations in these genes contributes to EOAD ranges from 17–80% depending on the examined population and the diagnostic criteria [1, 2]. Furthermore, the frequency is suggested to be even lower in the Swedish population [24]. Sequencing DNA from patients to identify new candidate genes has become more feasible the last decade with the emerging technology of next-generation massive-parallel sequencing. From whole-exome sequencing (WES) studies, variations in new candidate genes have been detected in different neurodegenerative diseases [10–12, 15, 18, 21]. Moreover, rare missense variants in the gene of the sortilin-related receptor 1, *SORL1*, have been shown to be enriched 1.5 fold in EOAD patients and the identification of pre-mature stop codons is restricted to patients only [26]. In this study, we applied WES on a family with inherited EOAD to identify a disease-causing mutation after having excluded mutations in *APP*, *PSEN1*, or *PSEN2*. Among six candidate variants, the *SORL1*-variant c.3907C > T was predicted to be likely pathogenic and was further characterized. Further *SORL1* gene-sequencing in 35 independent EOAD index cases as well as 183 EOAD cases, part of the reported European Early-Onset Dementia Consortium study [26], identified two additional *SORL1*-variants, c.3050-2A > G and c.5195G > C respectively, which segregated with disease. The clinical history of these three families and the immunohistochemical findings in brain tissue from two individuals from the WES-family are described. Taken together, this study has implications on how to review the pathogenic potential of *SORL1*-variants and provides information about histological and clinical evaluation of families with such variants.

## Material and methods

### Early onset AD cases, controls and DNA preparation

Three study cohorts, described below, were analyzed. DNA from patients diagnosed with AD and family-members were recruited at The Department of Geriatric Medicine, Karolinska University Hospital. DNA from controls were selected from the population-based study on persons over 60 years of age who live in the area of Kungsholmen, Stockholm, “The Swedish National Study on Aging and Care in Kungsholmen, SNAC-K” [14]. Peripheral blood was collected and genomic DNA was isolated using Gentra Puregene kit according to manufacturer’s protocol (Qiagen). Genomic DNA from formalin fixed paraffin embedded (FFPE) tissue, was isolated with the QIAamp® DNA FFPE tissue kit (Qiagen) according to manufacturer’s protocol. Mutations in *APP*, *PSEN1*, and *PSEN2*, as well as the repeat-expansion mutation in *C9orf72* were excluded prior to whole exome sequencing of PED.25, targeted re-resequencing of 35 EOAD cases and in all cases with *SORL1* variants in the case control study. The study was approved by the local ethics committee, Stockholm, and performed in harmony with the Helsinki Declaration with informed consents for all participants.

### Whole-exome sequencing

Whole-exome sequencing (WES) was performed on DNA from five siblings, four diagnosed with dementia and one unaffected, from a large family with inherited AD (PED.25). Sequencing was performed by the SNP & SEQ Technology platform in Uppsala, supported by the Swedish Council for Research Infrastructures and Uppsala University and hosted by the Science for Life Laboratory (SciLifeLab). The filtering performed on WES-data is schematically described in Table 1, and in detail as “Material and Methods” in the Additional file 1. The same filtering steps were applied on the variants in the targeted re-sequencing of candidate genes and on the variants generated from the EU EOD case-control study when applicable.

**Table 1:** Schematics of the filtering steps used for WES-data. The procedure used to find heterozygous candidate variants in whole-exome data from four affected siblings and one unaffected sibling in a family with early onset AD (PED.25)

Step	Criteria	Description	Number of variations passed	Genes with excluded variants (number of variants)
1	Rare and novel variants segregating with disease	CLC Genomic Workbench pipeline to extract shared variants in cases, not found in healthy sibling and with MAF < 1% in dbSNP138	1511	for gene names, see Additional file 5: Table S3
2	Non-synonymous or in splice-site region	Information annotated in CLC Genomic Workbench to include missense, nonsense, and variants in ± 2 nt of splice-site region	109	for gene names, see Additional file 5: Table S3 (1402)
3	Rare and novel variants in relevant populations	Excluded variants with MAF ≥ 1% found in either 1000G EUR, 1000G FIN, or in HBVDB Swedes/Danes	74	*RAD54L, MROH7, CENPF, TMEM63A, COCL6A6, TOPBP1, SLC17A4, TNXB, HLA-DPA1, CAPZA3, ABCC3, PRTN3, NOTCH2, PDE4DIP, PLEKHB2, MUC4, OR4C5, KCNJ12, TBC1D3, RP11-1407015.2, KRTAP-9, CDC27, EME1, SSC5D, C1orf94, XCR1, PIGZ, BTN1A1, TGFB2* (35)
4	Predicted to be deleterious, disease causing or to affect splicing	Alamut v.2.3 used for in-silico analysis of missense prediction to be either Deleterious (SIFT), to be Disease Causing (Mutation Taster) or being nonsense variations predicted to impact splicing by MaxEnt/NNSPLICE/HSF	45	*DMAP1, NBPF14, IGFN1, OBSCN, DNAH1, MUC4, TXNDC5, SLC17A3, MDC1, PRSS3, MUC6, PRR4, PRB1, C17orf74, CCDC144CP, MPP3, ZSCAN5A, SLC9B1P4, PLA2G3, C22orf42* (29)
5	Quality control of variant calls	Variants with low freq. (<20%), unbalanced F/R ratio (>0,1) and within repetitive sequence were removed	13	*SRGAP2, OBSCN, ANKRD36C, MUC4, FRG1, AP3S1, HLA-G, PFDN6, PRSS1, KMT2C, MUC6, SLC2A3, PRR4, PRB3, FAM186A, HNF1A, AC087499.7, RP11-1407O15.2, KRTAP1-3, KRTAP4-5, ANKFN1, ROCK1, CGB1, SPIB, FRG1B* (32)
6	Knowledge based prioritization	BioGSP to value expression profile and Ingenuity Pathway analysis to search for relevant processes	7	*PLSCR2, AOC2, AOC1, MYO7A, AQPR* (6)
7	Segregation analysis	Sanger sequencing in WES individuals and affected parent	6	*SLFNL1* (1)
8	Passed variations in genes:	*LTF, MME, FAM221A, UBE4A, SORL1, KDM2B*		

### Targeted re-sequencing of candidate genes

Genomic DNA from 35 index patients from families with early onset dementia were selected for screening of variants in the six candidate genes identified by WES in family PED.25. Of the 35 patients, 33 were diagnosed with AD whereas one was diagnosed with mixed vascular dementia and AD, and one with unspecified dementia. The mean age of onset was 57.5 ± 8.3 years in the index cases. An AmpliSeq custom gene-panel was designed for sequencing of the six genes, *LTF*, *MME*, *FAM221A*, *UBE4A*, *SORL1*, and *KDM2B* that targeted all coding regions including 10 bp of the flanking intronic regions. Detailed information of the procedure is found in the “Material and Methods” found in Additional file 1.

### Case-control study

As a part of the European Early-Onset Dementia (EU EOD) consortium, DNA from 183 AD cases with a mean age of onset at 58.4 ± 4.8 years and 303 healthy controls were screened for variations in *SORL1* [26]. The controls from the SNAC-K study were selected based on a Mini Mental State Examination (MMSE) score ≥ 28 (maximum score was 30), aged-matched (64.0 ± 5.4) and the absence of the following neurological diseases: frontotemporal dementia, semantic dementia, primary progressive aphasia/progressive non-fluent aphasia, corticobasal degeneration, progressive supranuclear palsy, Parkinson disease, multiple sclerosis, amyotrophic lateral sclerosis. In brief, all coding regions of *SORL1*, including 15 bp of flanking intronic regions were targeted by using Multiplex Amplification of Specific Targets for Resequencing (MASTR, www.multiplicom.com) technology followed by sequencing on a MiSeq platform (Illumina). Variant calling was made as described by Verheijen et al. [26].

### Sanger sequencing of genomic DNA and cDNA

Genomic DNA for all variants passing the in-house filtration step 5, Table 1, as well as additional family members included in the segregation analyses were Sanger-sequenced. Sequencing primers are available upon request. For sequencing of cDNA, total RNA was prepared from whole blood and cDNA was synthesized using Superscript® (Life Technolgies) with oligo (dT) primers. PCR was performed with primers positioned in *SORL1* exon 21, 5´-GCATATTCCGAGCTTCCAAA-3´, and exon 23, 5´-TCGCTCATGTCTCCACAGTC-3´. After PCR amplification, the products were separated on a 1.5% agarose gel and the bands around 200 bp (205 bp) and 400 bp (379 bp) were excised from the gel, purified with QIAquick® Gel Extraction Kit (Qiagen) and subjected to sequencing. All Sanger sequencing was performed using BigDye v.3.1 (Applied Biosystem Life technology) on ABI 3100/3500 instruments.

### Scoring pathogenicity of variants

The pathogenicity of the variants identified was classified in accordance with the guidelines of the American College of Medical Genetics and Genomics (ACMG) and the terminology recommended was used [22]. Note that the ACGM criteria are conservative and designed for use in clinical evaluation of gene variants in genes already known to cause monogenic forms of disease. In short, variants were considered as “likely benign” if two or more of the following criteria were fulfilled; variant is not segregating with disease, silent variant with no impact on splice-site, allele frequency greater than incidence of disease, not predicted by multiple *in silico* programs to affect protein. Variants were considered as “likely pathogenic” if three or more of the following criteria were fulfilled; variant only found in cases, variant segregates with disease (minimum three individuals, in two generations not being siblings), variant is predicted by two *in silico* programs to affect protein, variant changes the protein-length or is a loss-of-function mutation. Variants that could not be classified into either of these two were scored as “uncertain significance”.

### Immunohistochemical staining

Immunohistochemical (IHC) staining for detection of APP, Aβ and SORL1 were performed on 5 μm sections from formalin fixed paraffin embedded (FFPE)-tissue from cerebellum, frontal cortex and hippocampus from two post mortem AD cases from PED.25, four sporadic AD cases (no family history of dementia) and four controls (without pathological neurodegeneration). The sporadic AD cases and controls were matched to the two PED.25 family-members for age at death and gender, and Sanger-sequenced over the region of the PED.25 *SORL1*-variation, c.3907C > T, was performed to confirm a wild-type genotype. Staining with haematoxylin and eosin, luxol fast blue, Congo and Bielschowsky’s silver stain was performed according to standard procedures. Further immunohistochemical analysis of SORL1 was performed on hippocampal sections from ten additional sporadic AD cases and ten controls. For every staining occasion, one slide was stained without primary antibody (only antibody diluent in the primary antibody step) to serve as a negative control. Relevant positive controls were run in parallel. The slides were anonymized and evaluated semi quantitatively by two individuals independently. Specification of the antibodies used is available in Additional file 2: Table S1.

### Detection of interaction between SORL1 and APP in post-mortem brain using *in situ* proximity ligation assay

To specifically detect protein interaction between SORL1 and APP, Proximity Ligation Assay (PLA) analyses were performed on 5 μm sections from FFPE frontal cortex of human brain from two family members from PED.25, four sporadic AD cases and four controls previously described in the IHC section. The slides were deparaffinized and rehydrated prior to staining. Antigen retrieval was performed by boiling in DIVA (Biocare Medical) in a pressure cooker for 30 min at 110 °C. *In situ* PLA was performed using Brightfield detection reagents (OLINK Bioscience) according to the manufacturer’s instructions. The primary antibodies used were mouse anti-APP clone 10D1 (IBL 11090) diluted 1/1000 and rabbit anti-SORL1 clone epr14670 (Abcam ab190684) diluted 1/100, incubation overnight at +4 °C. The *in situ* PLA DNA-probes used were anti-mouse PLUS and anti-rabbit MINUS (OLINK, Bioscience). For every subject, two negative controls were performed by excluding one of the primary antibodies. One positive control for each antibody was performed by adding both PLUS and MINUS probe for the same antibody. Slides were scanned at 40× with a digital slide scanner (250 Flash Scanner, 3D Histech Ltd). Images of frontal cortex layer V were captured as TIFF images in Pannoramic Viewer (3D Histech Ltd). The TIFF images were opened in Duolink Image Tool (Olink, Bioscience) and all pyramidal neuronal cells were manually marked, and the number of PLA signals (APP-SORL1 interaction events) in each marked cell was counted by the system. A minimum of 10 images and 100 cells from each subject were analysed. The average number of PLA signals/neuron was compared between the groups.

## Results

### Whole-exome sequencing identifies *SORL 1* c.3907C > T, (p.Arg1303Cys) in PED.25

**Fig. 1:**
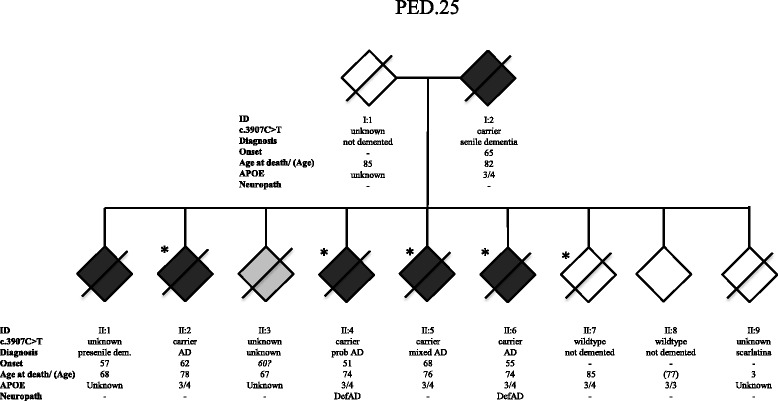
Pedigree of family PED.25. The family segregates the *SORL1* variant c.3907C > T (p.Arg1303Cys) in two generations. Individuals included in the whole-exome sequencing (WES) are marked with an *. The genetic status for variant c.3907C > T is indicated “carrier” for heterozygotes, “wildtype” when absent, and “unknown” if DNA was unavailable. Diagnosis refers to the clinical diagnosis. Onset refers to first observation of dementia symptoms, and *APOE* indicates the ε-alleles. Neuropath indicates the post mortem neuropathological diagnosis. The age at last known affection status of individuals still alive is indicated in parenthesis

PED.25 is a two-generational early onset AD family where seven family members from two generations were affected with dementia (Fig. 1). The mean age (± standard deviation) at onset of symptoms was 59.8 ± 5.9 years and the mean age of death was 74.1 ± 5.3 years. The mean duration of illness was 14.4 ± 5.9 years. Common initial symptoms were memory impairment, concentration difficulties, disorientation, visuospatial deficits, depression and aggressiveness. Later clinical features were lack of insight, apraxia and neuropsychiatric symptoms. Five family members developed severe dysphasia, three of them described as mute. Three suffered from rigidity or other symptoms of parkinsonism. Epileptic seizures were reported in two cases. Of the seven affected individuals, three received an AD diagnosis, one AD mixed type, one pre-senile dementia, one senile dementia and the last one was never evaluated in a clinical setting but had clear signs of dementia before death. Two of the cases with a clinical AD diagnosis were subjected to autopsy, and the neuropathological examination fulfilled the CERAD criteria for definitive AD and Braak stages V-VI. Cerebral amyloid angiopathy was also observed. Whole-exome sequencing (WES) on four siblings with AD and one healthy control sibling from PED.25, identified a total of 117 548 variants. Sequential filtering as outlined in Table 1 resulted in identification of a *SORL1* c.3907C > T, (p.Arg1303Cys) missense variation, Table 2. The *SORL1* variant was verified to be present in DNA extracted from FFPE tissue of the affected parent in the first generation. Combining the *in silico* predicted deleterious effect by SIFT and the disease-causing effect using Mutation taster, the low allele frequency of c.3907C > T in the Exome Aggregation Consortium database (0.006% [4/66720] in European Non-Finnish population) and the segregation data (present in 5 affected family members in 2 generations and absent in one unaffected) fits an autosomal dominant inheritance pattern of AD. Thus, the *SORL1* c.3907C > T, (p.Arg1303Cys) variant is scored as “likely pathogenic”. All five genotyped affected individuals carrying the *SORL1* c.3907C > T variant had the genotype *APOE* ε3/ε4 and the unaffected sibling was *APOE* ε3/ε3. Five additional variants, in the genes Lactotransferrin (*LTF*), Membrane metallo-endopeptidase (*MME*), Family with sequence similarity 221 Member A (*FAM221A*), Ubiquitination Factor E4A (*UBE4A*), and Lysine (K)-specific demethylase 2B (*KDM2B*) were also identified in the WES-filtration, see Additional file 3: Table S2 and the Additional file 1.

**Table 2:** *SORL1* variations. All *SORL1* variations found to be likely pathogenic or of uncertain significance according to ACMG criteria [25] generated using three independent study cohorts

Chr:pos hg19	Domain	Transcript Variant	Coding effect	Protein change	Predictions SIFT/MutTas	Prediction Splice-site effect MaxEnt/NNSPLICE/HSF	dbSNP ID	ExAC European Non-Finnish %	MAF % Case/Control EU EOD Swedes	Segregates with disease Family number	Classification ACMG	Study cohort
11:121340732	VPS10p	c.302C > T (NM_003105.5)	Missense	p.Ser101Phe	Deleterious/Disease causing	−1.3%	–	–	0.26/0	Unknown FH	Uncertain sign.	case-control
11:121383766	VPS10p	c.994C > T (NM_003105.5)	Missense	p.Arg332Trp	Deleterious/Disease causing	None	rs772110877	0.01049	0.26/0	Unknown FH	Uncertain sign.	case-control
11:121391400	VPS10p	c.1246C > T (NM_003105.5)	Nonsense	p.Arg416*	not applicable	−0.1%	rs144585461	–	0.26/0	Unknown FH ^a^	Likely path.	case-control
11:121437647	EGF	c.3050-2A < G (NM_003105.5)	Deletion	p.Gly1017-Glu1074del	not applicable	−100%	–	–	0/0	yes PED.27	Likely path.	Targeted re-seq.
11:121458821	LDLR class A	c.3907C > T (NM_003105.5)	Missense	p.Arg1303Cys	Deleterious/Disease causing	None	rs781023219	0.005995	0/0	yes PED.25	Likely path.	WES-family
11:121460051	LDLR class A	c.4030 T > C (NM_003105.5)	Missense	p.Cys1344Arg	Deleterious/Disease causing	None	–	–	0.26/0	Possible FH ^b^	Uncertain sign.	case-control
11:121478841	Fibronectin type III	c.5195G > C (NM_003105.5)	Missense	p.Gly1732Ala	Deleterious/Disease causing	None	rs777194720	0.007499	0.26/0	yes PED.1499	Uncertain sign.	case-control

*FH* Family history

^a^ Originates from Chile

^b^ Originates from Finland

### Targeted re-sequencing identifies *SORL1* splice variation c.3050-2A > G in PED.27

**Fig. 2:**
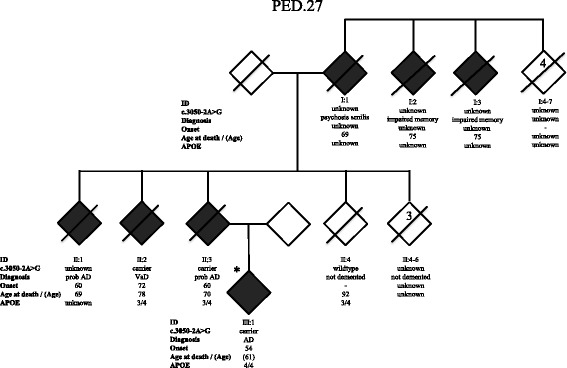
Pedigree of family PED.27. The family segregates the *SORL1* variant c.3050-2A > G (p.Gly1017-Glu1074) in two generations. Index patient (III:1) included in the targeted gene sequencing is marked with an *. The numbers inside the symbol for I:4–7 (4) and in II:4–6 (3) indicates multiple individuals. The genetic status for variant c.3050-2A > G is indicated “carrier” for heterozygotes, “wildtype” when absent, and “unknown” if DNA was unavailable. Diagnosis refers to the clinical diagnosis. Onset refers to first observation of dementia symptoms, and *APOE* indicates the ε-alleles. Diagnosis refers to the clinical diagnosis

**Fig. 3:**
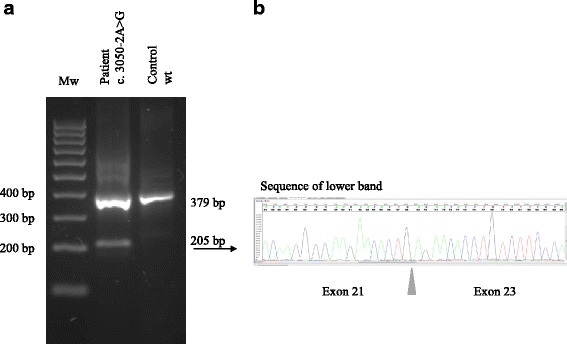
Sequencing of exons 21 to 23 of *SORL1* performed on cDNA. **a** Agarose gel of PCR products from a patient (PED.27 II:1) with the c3050-2A > G variant and a control (wt) showing the existence of two band in the patient, sized 205 bp and 379 bp, and only the larger 379 bp-band in the control, wild type individual. **b** Sequencing of the lower band in the c3050-2A > G carrier shows skipping of exon 22 in *SORL1*

Targeted re-sequencing of the six candidate genes (*LTF*, *MME*, *UBE4A SORL1, FAM221A* and *KDM2B*) was performed in 35 EOAD cases with a family history of dementia. A *SORL1* c.3050-2A > G variant was found in a patient with onset of AD at 54 years who belongs to a three-generational family with memory impairment, PED.27, (Fig. 2). In the parental generation, three subjects developed dementia including the index patient’s parent. The mean age at symptom onset for these four cases from two generations is 61.5 ± 7.5 years and the mean age of death among three deceased is 72.3 ± 4.9 years. Initial symptoms included impaired memory and visuospatial deficits. Three of four family members were reported to have early neuropsychiatric symptoms such as apathy, aggressiveness and hallucinations. They seem to have preserved their language skills and dysphasia was not a prominent symptom. Motor dysfunction with extrapyramidal symptoms such as rigidity, tremor and dystonia were seen in two cases. Two family members were diagnosed with probable AD, one with early onset AD and one with vascular dementia (VaD), although this VaD patient was argued to be a possible case of mixed AD because of visuospatial deficits. There were three additional relatives in an earlier generation who were described to have impaired memory or senile psychosis according to relatives’ description and or incomplete medical journals. DNA was available from a total of three affected family members and one unaffected individual who died at age 92 without signs of dementia. Segregation analysis for *SORL1* c.3050-2A > G confirmed segregation with disease. The *SORL1* c.3050-2A > G variant was predicted by in silico programs (MaxEnt, NNSPLICE and HSF) to have a detrimental effect on splicing leading to the loss of a splice-acceptor site at the nearest natural junction. The *SORL1* c.3050-2A > G variant is located two nucleotides upstream of the exon 22 acceptor site and to functionally test the effect on splicing, cDNA from individual II:3 in PED.27 was analyzed (Fig. 3). Amplification across exon 22 of the cDNA showed two distinct bands migrating close to the *in-silico* predicted sizes of 379 nucleotides for the wild-type mRNA, and 205 nucleotides for a transcript lacking exon 22, in agreement with exon-22 skipping i.e., a splicing effect in vitro (Fig. 3). Gel-extraction and re-sequencing of the lower band confirms the complete absence exon 22 (exon-skipping) in RNA obtained from the *SORL1* c.3050-2A > G carrier (Fig. 3). Furthermore, sequencing of the gel-purified, larger 379 bp band, which may appear stronger than the shorter band, shows the presence of both the wt and the deleted cDNA, indicating that the band contains heteroduplexes of the wt and the deleted cDNA. The effect at protein-level is an in-frame deletion of amino acids 1017–1074 (p.Gly1017-Glu1074del), removing the complete Epidermal growth factor-like (EGF) domain of SORL1. The *SORL1* c.3050-2A > G variant is thus scored as “likely pathogenic”.

Two of the affected and one unaffected family member were *APOE* ε3/ε4 carriers whereas the index case was an *APOE* ε4/ε4 carrier and had the earliest onset. Two additional variants in two separate index cases were identified in the targeted resequencing: one missense variant in *FAM221* and one missense variant in *SORL1* but none of these segregated with the disease in their respective families (Additional file 3: Table S2) and were thus scored as “likely benign”.

### Further characterization of case-control data identifies *SORL1* c.5195G > C in PED.1499

**Fig. 4:**
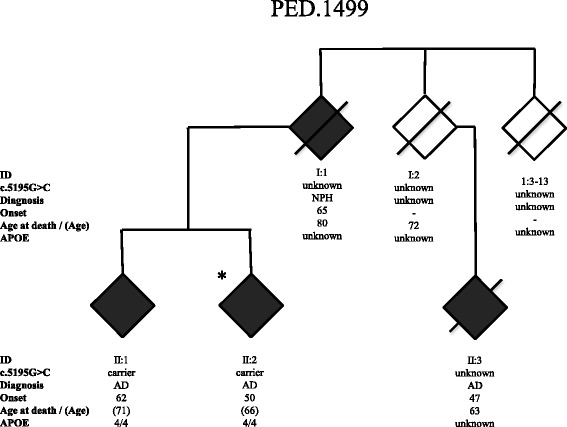
Pedigree of family PED.1499. The family segregates the *SORL1* variant c.5195G > C (p.Gly1732Ala) in one generation. Individual (II:2) included in the EU EOD case-control study is marked with an *. The genetic status for variant c.5195G > C is indicated “carrier” for heterozygotes, “wildtype” when absent, and “unknown” if DNA was unavailable. NPH = normal pressure hydrocephalus. Onset refers to first observation of dementia symptoms, and *APOE* indicates the ε-alleles. The age at last known affection status of individuals still alive is indicated in parenthesis

As described previously, DNA from 183 AD cases and 303 healthy controls, were screened for variations in the *SORL1* gene and constituted the case-control cohort from Sweden in a recent European Early-Onset Dementia (EU EOD) consortium study [26]*.* Applying the in-house filtering steps 1–5 presented in Table 1 on the *SORL1* sequencing data from the 183 AD cases, identifies 10 different variants of which five are predicted to be “likely benign”, (Additional file 3: Table S2). One variant, c.1246C > T, is a nonsense variant introducing a pre-mature stop codon p.Arg416* resulting in a truncated protein and is thus predicted to be “likely pathogenic” [26]. Unfortunately, no additional information on family history was available. Four of the identified variants were missense variations (p.Ser101Phe, p.Arg322Trp, p.Cys1344Arg, and p.Gly1732Ala) predicted be deleterious and disease causing using *in silico* SIFT and Mutation Taster programs respectively, (Table 2). No information on the family history was available for these cases except for p.Gly1732Ala.The *SORL1* missense variant c.5195G > C (p.Gly1732Ala) was detected in a case with a positive family history, PED.1499, (Fig. 4). In the Swedish family of PED.1499, four family members from two generations were affected with dementia (Fig. 4). The mean age of symptom onset was 56.0 ± 8.8 years and the mean age of death among two deceased was 71.5 ± 12.0 years. Three family members were diagnosed with AD. Common initial symptoms were memory impairment, visuospatial deficits, anxiety and depression. One of the family members in the first generation presented with simultaneous incontinence and gait disorder and was subsequently diagnosed with normal pressure hydrocephalus (NPH). The NPH patient temporarily improved upon surgical implantation of a ventriculoperitoneal shunt, but memory function continued to deteriorate. Other clinical features in the family were apraxia, neuropsychiatric symptoms and hallucinations. Structural neuroimaging (MRI) showed mild to moderate hippocampal atrophy and mild cortical atrophy, but also atypical findings of small hemorrhages in the parieto-temporal and temporo-occipital areas in two cases. DNA was available from two cases with AD in the second generation and sequencing confirmed segregation of the *SORL1* missense variant c.5195G > C in both cases. They were both homozygous *APOE* ε4/ε4 carriers. The variant was not present in any of the Swedish control cases and the allele frequency in the ExAC database was 0.007% in non-Finnish Europeans. The combined information available for *SORL1* c.5195G > C is not enough to be classified as “likely pathogenic” according to the ACMG criteria and it is thus scored as “uncertain significance”.

### Neuropathology and immunohistochemistry

**Fig. 5:**
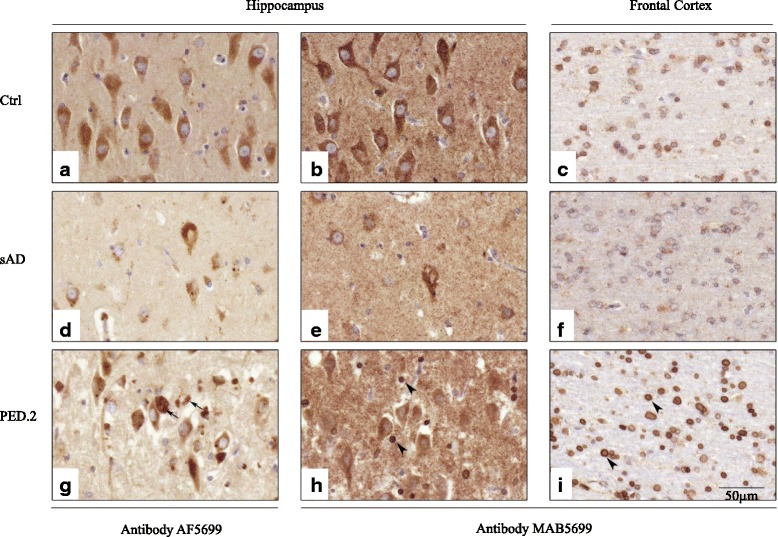
Immunohistochemical localization of SORL1 in postmortem brain material from controls, sporadic AD and PED.25. Representative pictures from control (**a**-**c**), sporadic AD (**d**-**f**) and PED.25 (**g**-**i**) in the CA1 region of hippocampus (**a**-**b**, **d**-**e**, **g**-**h**) and subcortical white matter in frontal cortex (**c**, **f**, **i**) using two different SORL1 antibodies, AF5699 (**a**, **d**, **g**) and MAB5699 (**b**-**c**, **e**-**f**, **h**-**i**). The AF5699 SORL1 antibody showed an intense immunoreactivity of extracellular SORL1 aggregates in PED.25 (*arrows* in **g**). Arrowheads indicate strong SORL1 immunoreactivity (MAB5699) in glial cells in *grey* (**h**) and *white* matter (**i**) in the affected member from PED.25. Scalebar: 50 μm. Ctrl = control, sAD = sporadic AD, PED.25 = affected family member II:6

Post-mortem brain tissue was available from two of the affected family members from PED.25 (II:4 and II:6, Fig. 1), and in both cases a definite diagnosis of AD was established in accordance with CERAD criteria and Braak stages V-VI [4]. Numerous neuritic and diffuse plaques immunoreactive for Aβ were observed in frontal cortex and hippocampus in the two cases from PED.25 and the morphology and number of plaques were similar to sporadic AD cases, unlike the controls where no amyloid plaques were detected (data not shown). Since a candidate variation in PED.25 was located in the *SORL1* gene (p.Arg1303Cys) we characterized the expression pattern of SORL1 in the two affected family members and compared it to sporadic AD cases and controls using immunohistochemistry with four different SORL1 antibodies (Additional file 2: Table S1). As previously reported by Dodson et al. [9], SORL1 immunoreactivity in control cases was characterized by a punctate staining pattern mainly in the somatodendritic compartment of pyramidal neurons in frontal cortex and hippocampus and it was confirmed with all four SORL1 antibodies used in this study (Fig. 5, a-c; and Additional file 4: Figure S1). A semiquantitative evaluation of the SORL1 immunoreactivity in the pyramidal neurons in frontal cortex and hippocampus showed that there is a reduction in the two PED.25 affected family members compared to controls (*n* = 4) and to sporadic AD cases (*n* = 4). The staining is most noticeable in neurons using MAB5699 or 612633 and in hippocampus when staining with ab190684 (see Additional file 4: Figure S1). However, the most striking observation was the strong immunoreactivity with the MAB5699 SORL1 antibody in glial cells of grey and white matter in the two affected family members (Fig. 5, h-i). Glial staining is not seen with any of the other antibodies used (see Additional file 4: Figure S1). This immunoreactivity in glial cells was also seen to a certain extent in one of the four sporadic AD cases. Furthermore, using an antibody towards the VPS10P-domain of SORL1, AF5699, the two PED.25 cases showed a striking immunoreactivity to SORL1 aggregates in hippocampus CA1 that was located extracellularly (Fig. 5, g). To evaluate if the atypical SORL1 immunoreactivity patterns were exclusively found in PED.25 family members, we analyzed 10 additional sporadic AD cases and 10 controls, and found that in total five out of 14 sporadic AD cases and two of the 14 controls showed some immunoreactivity in glial cells, similar to the pattern in the two PED.25 cases. In addition, SORL1 extracellular immunoreactive aggregates could be detected to some extent in five of 14 sporadic AD cases and in two of the 14 controls. In summary, we found that the two affected cases in PED.25 showed an atypical SORL1 staining pattern that has not been described before, and that this pattern was also found to some extent and less pronounced in some sporadic AD cases and controls.

### *In situ* proximity ligation assay

**Fig. 6:**
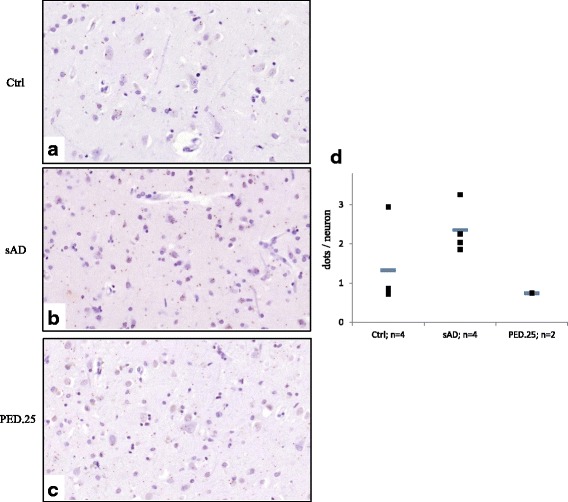
Co-localization between SORL1 and APP in postmortem brain as detected by *in situ* PLA. **a**-**c** shows representative sections of the three groups; **a** Ctrl = controls, **b** sAD = sporadic AD, and **c** PED.25 = affected family member from PED.25. **d** The mean number of PLA dots/neuron, represented as horizontal bars (**−**), was quantified in pyramidal neurons in frontal cortex from controls (*n* = 4), sporadic AD (*n* = 4) and PED.25 (*n* = 2). A minimum of 100 neurons was quantified from every individual. The individual values are represented as filled squares (■)

In order to study if the SORL1 p.Arg1303Cys variation affects the binding of SORL1 to APP, we used the *in situ* proximity ligation assay, PLA, to quantify the co-localization between the two proteins in pyramidal neurons of the frontal cortex. This was achieved by using a SORL1 antibody, ab190684, directed towards the LDLR class A repeat region previously reported to be necessary for interaction with APP [17]*.* In control individuals (*n* = 4), the co-localization between SORL1 and APP was low in all individuals but one. The mean ± standard deviation PLA-signal per neuron was calculated to be 1.32 ± 1.08 (Fig. 6) in the controls, whereas in the sporadic AD cases (*n* = 4) the PLA-signal per neuron was 2.35 ± 0.63. The two affected cases in PED.25 (*n* = 2) presented a low PLA-signal per neuron, 0.74 ± 0.01, suggesting similar levels of co-localization of APP and SORL1 as seen in the controls, but different from sporadic cases of AD (Fig. 6). No apparent differences in the subcellular localization of the PLA-signal were observed between the three groups.

## Discussion

Using three different patient cohorts of early onset Alzheimer disease, we describe three *SORL1* variants that segregate with disease in three families adding to the literature which supports *SORL1* as a major player in AD pathoetiology. *SORL1*, was initially identified as a risk-factor in a case-control association-study [23], but has more recently been implicated in familial early-onset as well as late-onset AD [8, 16, 20, 21, 25, 26].

Applying WES, we identified the first variant, *SORL1* c.3907C > T, (p.Arg1303Cys), in a two generational family with neuropathologically confirmed early onset AD (PED.25). The evidence for the variant to be considered as causative is the combined criteria of, *in silico* prediction of the missense substitution, the frequency of the variation in population databases, and the pattern of segregation. Collectively, this classifies the variation p.Arg1303Cys as “likely pathogenic”. Notably, the variant p.Arg1303Cys segregating in PED.25 (Fig. 1) is located in the central part of the LDLR class A-domain, a region that has been demonstrated to be crucial for binding SORL1 to APP and reduce Aβ-production [17]. The potential effect of SORL1 p.Arg1303Cys, on human post mortem brain pathology was explored by IHC staining on two autopsy cases from PED.25. The staining was largely made as an initial screening for understanding how to proceed with future IHC staining and knowledge of the properties of the available commercial antibodies (Additional file 2: Table S1). Semiquantitative analysis demonstrated a general reduction of SORL1-staining in pyramidal neurons in frontal cortex and hippocampus in p.Arg1303Cys carriers compared with both controls and sAD. We also found that the antibody AF5699 directed against the Aβ-binding VPS10P-domain [6], resulted in strong staining of seemingly extracellular SORL1 aggregates, while the MAB5699 antibody showed strong immunoreactivity in glial cells both in grey and white matter. These atypical staining patterns were found in both the two p.Arg1303Cys carriers, as well as in some of the sporadic AD and in a few control cases although with weaker intensity. The sAD cases with immunoreactivity in glia and extracellular aggregates appeared to overlap in sAD (4/5) but not in the controls. It is plausible that this indicates that the p.Arg1303Cys variant affects the SORL1 functionality in brain. Since the p.Arg1303Cys variation is located in a region of SORL1 that is important for interaction with APP, we next performed a pilot test to investigate co-localization of SORL1 and APP by the use of PLA. We found no difference in SORL1/APP interactions between controls and affected members from PED.25, which could mean that the p.Arg1303Cys variation at least to some extent preserves the binding to APP. On the other hand, since the sporadic AD cases have an elevated interaction between SORL1 and APP, the variant in PED.25 could also destabilize the interaction between APP and SORL1. Both the IHC staining pattern and the PLA experiments indicate that there are in fact differences between the p.Arg1303Cys carriers compared with controls and sporadic AD cases but these need to be investigated in larger patient series and more detail to draw any pathogenic conclusions. It will also be valuable to extend the analysis to include carriers with mutations in *APP*, *PSEN1* or *PSEN2*.

**Fig. 7:**
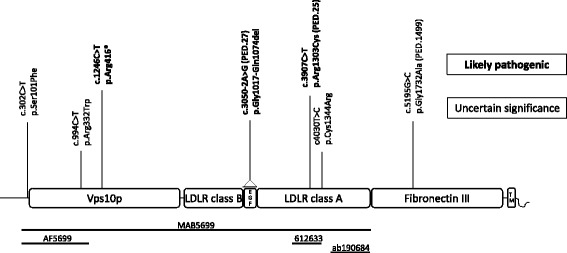
Schematic picture of the SORL1 protein adapted from Verheijen et al., presenting the location of *SORL1* variants that are “likely pathogenic” (in bold) and variants of “uncertain significance”, also see Table 2. Functional domains are based on Uniprot information (Q92673), and numbering of variations are based on NM_003105.5 (cDNA) and NP_003096 (protein). Below are lines corresponding to the epitopes used to generate the respective antibodies (MAB5699, AF5699, 612633 and ab190684) applied in the study. Vps10p: vacuolar protein sorting 10 domain; LDLR class B: low-density lipoprotein-receptor class B repeats; EGF: epidermal growth factor-like domain; LDLR class A: low-density lipoprotein-receptor class A domain; Fibronectin III: Fibronectin type-III domain; TM: transmembrane domain

The second variant, *SORL1* c.3050-2A > G, segregated with disease in family PED.27 in three individuals from two generations and was absent in one healthy control, who passed away at the age of 92 year (Fig. 2). This intronic variant causes exon 22-skipping that results in a deletion of amino acids Gly1017-Glu1074 of the protein (Fig. 3). The deletion removes the EGF domain of SORL1 that lies in between the LDLR class B and the LDLR class A domains and it is likely that it would not only make the protein shorter but also affect its structure and/or function (Fig. 7). It is not possible to quantify the relative amounts of the deleted and the wt transcripts from the agarose gel results because the PCR conditions were not quantitative and the non-denaturing conditions of the gel electrophoresis allows for formation of heteroduplexes between the wt and the deleted cDNA which both migrate at the approximate size of the wt (379 bp). Previous studies have suggested that haploinsuffienciency may be a mode of action for the *SORL1* variants associated with AD [26] and it is possible that the SORL1 c.3050-2A > G with loss of exon22 would result in such an effect but more studies are required to understand the mode of action. The evidence for the variant to be considered as causative is the combined criteria of; the change in protein length, the frequency of the variation in population databases, and the pattern of segregation. Collectively, this classifies the variation p.Gly1017-Glu1074del as “likely pathogenic”.

The third variant described, *SORL1* c.5195G > C (p.Gly1732Ala) was first identified in the case-control study reported in Verheijen 2016 [26]. The index case belongs to a family with early onset AD and the variant was detected in two siblings in PED.1499. This variation is in the fibronectin type III domain of SORL1, and a possible functional impact is not known. Although the *in silico* prediction is deleterious and disease causing and the allele frequency in the European population is less than 0.01%, the low number of family members in the segregation analyses will not allow the variant to be scored as “likely pathogenic” according to the criteria of ACMG and the variant is thus classified to be of “uncertain significance”. Follow-up studies in PED.1499 may lead to additional cases in the family.

Our clinical descriptions of the affected individuals from the three families provide information about possible shared phenotypes and symptoms in AD that could be explained by variations in the *SORL1*-gene. All three families have relatively homogenous features, sharing AD as the main clinical diagnosis and initial symptoms such as memory impairment and visuospatial deficits. Since *APOE*-ε4 alleles are suggested to modify the effects of *SORL*-gene variations on Aβ-processing [16], we provided the genotypes on all subjects in the families but the low number of cases in this study makes it impossible to make any statistical inferences or conclusions on *APOE*’s possible modifying effect on the phenotype at this point.

It is unclear why the discovery of additional monogenic causes of familial AD has been more or less arrested since the 1990’s. Furthermore, the reported *SORL1* families have so far been significantly smaller than the original FAD families. Variable disease onset, variable expression i.e., heterogeneous phenotypes and or reduced penetrance, private very rare mutations and phenocopies are plausible explanations which may mask an autosomal dominant inheritance. It is likely that the use of whole exome and whole genome sequencing in smaller families will be an effective tool for new and rare gene discoveries but nevertheless thorough genotype-phenotype studies will be essential both for elucidating possible disease modifiers such as *APOE* and for understanding the penetrance of for example *SORL1* variants.

## Conclusions

The genetic findings of three different *SORL1* variants that segregate in three families with inherited AD strengthen the likely pathogenic nature of *SORL1.* Furthermore, the IHC staining, together with the described clinical features of the families, will be valuable for the continued evaluation of *SORL1* as a monogenic cause of familial AD. Future studies of additional families and follow-up studies in the presented families will be important to finally conclude upon the importance of *SORL1* in AD.

## Additional files

Additional file 1: Material and Methods. (DOCX 23.9 kb)

Additional file 2: Table S1. Specification of the antibodies used in the immunohistochemical investigation. (DOCX 13 kb)

Additional file 3: Table S2. All variants fulfilling the filtration criteria in the three study cohorts; WES-family, targeted re-sequencing, and case-control study. (DOCX 18 kb)

Additional file 4: Figure S1. Immunohistochemical localization of SORL1 in postmortem brain material. Representative pictures from control, sporadic AD and from two individuals from PED.25 in the CA1 region of hippocampus using four different SORL1 antibodies, AF5699 (a-d) and MAB5699 (e-h), 612633 (i-l) and ab190684 (m-p). Scalebar: 100 μm. Ctrl = control, sAD = sporadic AD, PED.25 II-4= affected family member II:4 and PED.25 II-6= affected family member II:6. (ZIP 36668 kb)

Additional file 5: Table S3. The 1511 gene variants identified through filtering of WES data step 1 (rare and novel variants present in 4 affected and absent in 1 unaffected) in PED.25 with chromosomal position and gene annotation. (XLSX 81 kb)

## Abbreviations

AD: Alzheimer disease
APP: Amyloid beta precursor protein
Aβ: Amyloid beta
EOAD: Early-onset Alzheimer disease
FFPE: Formalin fixed paraffin embedded
MAF: Minor allele frequency
PSEN1: Presenilin 1
PSEN2: Presenilin 2
SORL1: Sortelin-related receptor-1
WES: Whole-exome sequencing

## References

[1] Arango D, Cruts M, Torres O, Backhovens H, Serrano ML, Villareal E, Montanes P, Matallana D, Cano C, Van Broeckhoven C (2001). Systematic genetic study of Alzheimer disease in Latin America: mutation frequencies of the amyloid beta precursor protein and presenilin genes in Colombia. Am J Med Genet.

[2] Avramopoulos D (2009). Genetics of Alzheimer's disease: recent advances. Genome Med.

[3] Bertram L, Lill CM, Tanzi RE (2010). The genetics of Alzheimer disease: back to the future. Neuron.

[4] Braak H, Braak E (1991). Neuropathological stageing of Alzheimer-related changes. Acta Neuropathol.

[5] Cacace R, Sleegers K, Van Broeckhoven C (2016). Molecular genetics of early-onset Alzheimer's disease revisited. Alzheimers Dement.

[6] Caglayan S, Takagi-Niidome S, Liao F, Carlo AS, Schmidt V, Burgert T, Kitago Y, Fuchtbauer EM, Fuchtbauer A, Holtzman DM (2014). Lysosomal sorting of amyloid-beta by the SORLA receptor is impaired by a familial Alzheimer's disease mutation. Sci Transl Med.

[7] Cruts M, Theuns J, Van Broeckhoven C (2012). Locus-specific mutation databases for neurodegenerative brain diseases. Hum Mutat.

[8] Cuccaro ML, Carney RM, Zhang Y, Bohm C, Kunkle BW, Vardarajan BN, Whitehead PL, Cukier HN, Mayeux R, St George-Hyslop P (2016). SORL1 mutations in early- and late-onset Alzheimer disease. Neurol Genet.

[9] Dodson SE, Gearing M, Lippa CF, Montine TJ, Levey AI, Lah JJ (2006). LR11/SorLA expression is reduced in sporadic Alzheimer disease but not in familial Alzheimer disease. J Neuropathol Exp Neurol.

[10] Freischmidt A, Wieland T, Richter B, Ruf W, Schaeffer V, Muller K, Marroquin N, Nordin F, Hubers A, Weydt P (2015). Haploinsufficiency of TBK1 causes familial ALS and fronto-temporal dementia. Nat Neurosci.

[11] Guerreiro RJ, Lohmann E, Bras JM, Gibbs JR, Rohrer JD, Gurunlian N, Dursun B, Bilgic B, Hanagasi H, Gurvit H (2013). Using exome sequencing to reveal mutations in TREM2 presenting as a frontotemporal dementia-like syndrome without bone involvement. JAMA Neurol.

[12] Guerreiro RJ, Lohmann E, Kinsella E, Bras JM, Luu N, Gurunlian N, Dursun B, Bilgic B, Santana I, Hanagasi H (2012). Exome sequencing reveals an unexpected genetic cause of disease: NOTCH3 mutation in a Turkish family with Alzheimer's disease. Neurobiol Aging.

[13] Harvey RJ, Skelton-Robinson M, Rossor MN (2003). The prevalence and causes of dementia in people under the age of 65 years. J Neurol Neurosurg Psychiatry.

[14] Lagergren M, Fratiglioni L, Hallberg IR, Berglund J, Elmstahl S, Hagberg B, Holst G, Rennemark M, Sjolund BM, Thorslund M (2004). A longitudinal study integrating population, care and social services data. The Swedish National study on Aging and Care (SNAC). Aging Clin Exp Res.

[15] Logue MW, Schu M, Vardarajan BN, Farrell J, Bennett DA, Buxbaum JD, Byrd GS, Ertekin-Taner N, Evans D, Foroud T (2014). Two rare AKAP9 variants are associated with Alzheimer's disease in African Americans. Alzheimers Dement.

[16] Louwersheimer E, Cohn-Hokke PE, Pijnenburg YA, Weiss MM, Sistermans EA, Rozemuller AJ, Hulsman M, van Swieten JC, van Duijn CM, Barkhof F (2017). Rare genetic variant in SORL1 may increase penetrance of alzheimer's disease in a family with several generations of APOE-varepsilon4 homozygosity. J Alzheimers Dis.

[17] Mehmedbasic A, Christensen SK, Nilsson J, Ruetschi U, Gustafsen C, Poulsen AS, Rasmussen RW, Fjorback AN, Larson G, Andersen OM (2015). SorLA complement-type repeat domains protect the amyloid precursor protein against processing. J Biol Chem.

[18] Menezes MP, Waddell L, Lenk GM, Kaur S, MacArthur DG, Meisler MH, Clarke NF (2014). Whole exome sequencing identifies three recessive FIG 4 mutations in an apparently dominant pedigree with Charcot-Marie-Tooth disease. Neuromuscul Disord.

[19] Mirra SS, Heyman A, McKeel D, Sumi SM, Crain BJ, Brownlee LM, Vogel FS, Hughes JP, van Belle G, Berg L (1991). The Consortium to Establish a Registry for Alzheimer's Disease (CERAD). Part II. Standardization of the neuropathologic assessment of Alzheimer's disease. Neurology.

[20] Nicolas G, Charbonnier C, Wallon D, Quenez O, Bellenguez C, Grenier-Boley B, Rousseau S, Richard AC, Rovelet-Lecrux A, Le Guennec K (2015). SORL1 rare variants: a major risk factor for familial early-onset Alzheimer's disease. Mol Psychiatry.

[21] Pottier C, Hannequin D, Coutant S, Rovelet-Lecrux A, Wallon D, Rousseau S, Legallic S, Paquet C, Bombois S, Pariente J (2012). High frequency of potentially pathogenic SORL1 mutations in autosomal dominant early-onset Alzheimer disease. Mol Psychiatry.

[22] Richards S, Aziz N, Bale S, Bick D, Das S, Gastier-Foster J, Grody WW, Hegde M, Lyon E, Spector E (2015). Standards and guidelines for the interpretation of sequence variants: a joint consensus recommendation of the American College of Medical Genetics and Genomics and the Association for Molecular Pathology. Genet Med.

[23] Rogaeva E, Meng Y, Lee JH, Gu Y, Kawarai T, Zou F, Katayama T, Baldwin CT, Cheng R, Hasegawa H (2007). The neuronal sortilin-related receptor SORL1 is genetically associated with Alzheimer disease. Nat Genet.

[24] Thonberg H, Fallstrom M, Bjorkstrom J, Schoumans J, Nennesmo I, Graff C (2011). Mutation screening of patients with Alzheimer disease identifies APP locus duplication in a Swedish patient. BMC Res Notes.

[25] Vardarajan BN, Zhang Y, Lee JH, Cheng R, Bohm C, Ghani M, Reitz C, Reyes-Dumeyer D, Shen Y, Rogaeva E (2015). Coding mutations in SORL1 and Alzheimer disease. Ann Neurol.

[26] Verheijen J, Van den Bossche T, van der Zee J, Engelborghs S, Sanchez-Valle R, Llado A, Graff C, Thonberg H, Pastor P, Ortega-Cubero S (2016). A comprehensive study of the genetic impact of rare variants in SORL1 in European early-onset Alzheimer's disease. Acta Neuropathol.

