# Supplementary material for: Identification and description of three families with familial Alzheimer disease that segregate variants in the SORL1 gene
Source: Acta Neuropathol Commun. 2017 Jun 9;5:43. doi: 10.1186/s40478-017-0441-9 (PMC5465543; doi:10.1186/s40478-017-0441-9)
Supplement: Supplementary file 1 — Material and Methods. (DOCX 23.9 kb) [file 40478_2017_441_MOESM1_ESM.docx]

***Supplementary information***

**Identification and description of three families with familial Alzheimer disease that segregate variants in the *SORL1* gene**

**Håkan Thonberg^a,b^, Huei-Hsin Chiang^a,b^, Lena Lilius^a,b^, Charlotte Forsell^a,b^, Anna-Karin Lindström^a,b^, Charlotte Johansson^a,b^, Jenny Björkström^a,b^, Steinunn Thordardottir^a,b^, Kristel Sleegers^c,d^, Christine Van Broeckhoven^c,d^, Annica Rönnbäck^a,b^, Caroline Graff^a,b^**

^a^ Karolinska Institutet, Department NVS, Center for Alzheimer Research, Division for Neurogeriatrics, Huddinge, Sweden

^b^ Karolinska University Hospital, Department of Geriatric Medicine, Genetics unit, Stockholm, Sweden

^c^ Neurodegenerative Brain Disease Group, Department of Molecular Genetics, VIB, Antwerp, Belgium

^d^ Laboratory of Neurogenetics, Institute Born-Bunge, University of Antwerp, Antwerp, Belgium

**Content:**

**Material and Methods**

**Case Reports**

**Material and Methods**

**Whole-exome sequencing**

In brief, genomic DNA was prepared by sonication (Covaris). The exons were captured using TruSeq exome enrichment kit (Illumina Inc) and paired-end sequenced with 100 bp read length on Illumina HiSeq2000. Sequence reads were mapped to the reference genome hg19 using the Burrows-Wheeler aligner, SAM tools to generate BAM files, and GATK to generate joint variant calling files. The variant calls were then further processed using CLC Genomic Workbench (Qiagen) by applying in-house pipeline filtration steps to filter and annotate variants. Variants segregating with dementia, i.e. present as heterozygous variants in all four affected and absent in the healthy sibling, were kept and filtered to exclude common variants found in dbSNP 138 (MAF ≥0.01) (Table 1). Variants being either non-synonymous or located in a splice site region ±2 nt, thereby possibly affecting splicing, were kept as candidates. Further filtering was done by removing variants with a MAF ≥ 0.01 found in either one out of three public datasets; 1000G Eur, 1000G Fin, or in Human Background Variation DataBase Swedes/Danes (http://neotek.scilifelab.se/hbvdb/). In silico missense prediction and splice-site analyses were performed using Alamut v.2.3 for the remaining 74 variants and variants were kept that were predicted by at least one out of three algorithms either as “Deleterious” in SIFT, as “Disease causing” in Mutation Taster, or as to affect splice-site at nearest natural junction with an average greater than 20% from three in-silico prediction programs (MaxEnt, NNSPLICE, and HSF). Next, calls made in repetitive sequences or with low frequency calls (< 20%), as well as unbalanced forward/reverse ratio (> 0.1) were removed. A knowledge-based prioritization was made by scoring for the expression of the variant genes in the central nervous system (CNS) according to data available at BioGPS (<http://biogps.org/>). The scoring was based on the median expression for a gene in the dataset GeneAtlas U133A and involved taking the two highest expressed tissues in consideration and comparing it to the expression-levels found in tissues from the central nervous system. The prioritization also employed the use of Ingenuity Pathway Analysis, Gene View (Qiagen), to score the keywords; APP, β-amyloid, dementia or any other neurodegenerative diseases. In summary, all genes with a high expression in CNS were kept. Genes expressed relatively low in CNS, < 0.1 times the median expression level, were also kept if any of the key words were present in Ingenuity Pathway Analysis, Gene View (Qiagen). Lastly, in step 7 segregation was complemented by Sanger sequencing of DNA obtained from FFPE heart-tissue from the affected parent in family PED.25 and variants confirmed to be present were kept

**Targeted re-sequencing of candidate genes**

An AmpliSeq custom gene-panel was designed for sequencing of the six candidate genes, *LTF*, *MME*, *FAM221A*, *UBE4A*, *SORL1*, and *KDM2B*, that targeted all coding regions including 10 bp of the flanking intronic regions. Sequencing was performed on Ion Torrent PGM machine, on two Ion 316™ Chips, and post run report and analysis was performed with Ion Torrent Suite 4.6 at the NGI Uppsala, Uppsala Genome Center, SciLifelab, Sweden. The sequence reads were aligned to hg19 and the mean coverage over the 136 regions was 604X with a success rate of 96.7 % using a criterion of full coverage if >10X. Variant calling was made by applying an in-house pipe-line filtration using CLC Genomic Workbench (Qiagen). The variants were filtered to exclude common variants in dbSNP138 and variants in European subpopulations reaching MAF ≥ 0.01, as well as low quality calls, with coverage below 10X, or calls with an unbalanced forward/reverse ratio (>0.1). Also, calls made in regions with adjacent homopolymers were visually examined to evaluate their quality and specificity.

**Immunohistochemical staining**

Staining was performed on samples with standard methods. Briefly, the slides were deparaffinised and rehydrated prior to staining. After antigen retrieval, sections were blocked for endogenous peroxidase (Peroxidase Block, Dako) for 5 min at RT and incubated with 5% normal goat serum 20 min at RT, except for the slides with primary antibody made in sheep that were blocked with serum free protein block (Dako). The primary antibodies diluted in Antibody Diluent (Dako), were incubated on sections for 45 min at RT, followed by 30 min incubation with the secondary antibody at RT. The immunoreactions were visualized with DAB. All sections were counterstained with haematoxylin for 20 seconds and blued in tap water. The sections were washed thoroughly in Tris-buffered saline with 0.05% Tween® 20 (Sigma-Aldrich) between each step. Dehydration was performed with increasing concentration of ethanol (70%-99% ethanol), clearing in xylene and mounting with DPX mountant (VWR).
