# Supplementary material for: Identification and description of three families with familial Alzheimer disease that segregate variants in the SORL1 gene
Source: Acta Neuropathol Commun. 2017 Jun 9;5:43. doi: 10.1186/s40478-017-0441-9 (PMC5465543; doi:10.1186/s40478-017-0441-9)
Supplement: Supplementary file 2 — Specification of the antibodies used in the immunohistochemical investigation. (DOCX 13 kb) [file 40478_2017_441_MOESM2_ESM.docx]

| **Table S1. Specification of the antibodies used in the immunohistochemical investigation** | | | | | | | |
| --- | --- | --- | --- | --- | --- | --- | --- |
|  |  |  |  |  |  |  |  |
| **Antigen** | **Epitope** | **Prod no** | **Clone** | **Supplier** | **Dilution (µg/ml)** | **Antigen retrieval** | **Secondary antibody** |
| APP | N-terminal | 11090 | 10D1 | Tecan | 0.1 | DIVA^a^ | Envision Mouse (Dako K4007) |
| Aβ | amino acids 1-16 | SIG39320-200 | 6E10 | BioLegend | 0.5 | Formic acid^c^ | Envision Mouse |
| SORL1 | amino acids 1350-1550 | Ab190684 | Epr14670 | abcam | 3.5 | BORG^b^ | Envision Rabbit (Dako K4011) |
| SORL1 | amino acids 82-1550 | MAB5699 | 525122 | R&D systems | 7.5 | DIVA | Envision Mouse |
| SORL1 | amino acids 1220-1337 | 612633 | 48/LR11 | BD | 0.25 | BORG | Envision Mouse |
| SORL1 | amino acids 82-367 | AF5699 | polyclonal | R&D systems | 5 | DIVA | Donkey anti sheep HRP (AbD Serotec STAR88P) |
| ^a^ Biocare Medical DV2004MX incubated 30min at 110°C in a pressure cooker | | | | | | | |
| ^b^ Biocare Medical BD1000G1 incubated 30min at 110°C in a pressure cooker | | | | | | | |
| ^c^ 70% incubated 20 min at room temperature | | | | | | | |
