# Supplementary material for: Identification and description of three families with familial Alzheimer disease that segregate variants in the SORL1 gene
Source: Acta Neuropathol Commun. 2017 Jun 9;5:43. doi: 10.1186/s40478-017-0441-9 (PMC5465543; doi:10.1186/s40478-017-0441-9)
Supplement: Supplementary file 3 — All variants fulfilling the filtration criteria in the three study cohorts; WES-family, targeted re-sequencing, and case-control study. (DOCX 18 kb) [file 40478_2017_441_MOESM3_ESM.docx]

| **Table S2. All variants fulfilling the filtration criteria in the three study cohorts; WES-family, targeted re-sequencing, and case-control study** | | | | | | | | | | | | | |
| --- | --- | --- | --- | --- | --- | --- | --- | --- | --- | --- | --- | --- | --- |
| Chr:pos hg19 | Gene | Domain | Transcript Variant | Coding effect | Protein change | Predictions SIFT / MutTas | Prediction Splicesite effect  MaxEnt / NNSPLICE / HSF | dbSNP ID | ExAC  Europeans % | MAF %  Case / Control EU EOD Swedes | Segregates with disease  Family name | Classification ACMG | Study cohort |
| 3:46501155 | *LTF* | Transferrin-like | c.198G>C (NM_002343.4) | Missense | p.Gln66His | Tolerated / Disease caus. | None | rs763312524 | 0.012 (Non-Finnish) | Not applicable | yes  PED.25 | Uncertain sign. | WES-family |
| 3:154886403 | *MME* | Peptidase M13 | c.1903G>T (NM_000902.3) | Missense | p.Gly635Cys | Deleterious / Disease caus. | None | rs766507428 | 0.0060 (Non-Finnish) 0.015 (Finnish) | Not applicable | yes  PED.25 | Uncertain sign. | WES-family |
| 7:23731113^ 23731114 | *FAM221A* | not known | c.535insA (NM_199136.3) | Nonsense | p.Pro181Thrfs*29 | Not applicable^a^ | None | rs772334819 | 0.024 (Non-Finnish) 0.015 (Finnish) | Not applicable | yes  PED.25 | Uncertain sign. | WES-family |
| 7:23731156 | *FAM221A* | not known | c.5786G>C (NM_199136.4) | Missense | p.Gly193Ala | Deleterious / Disease causing | None | rs200212919 | 0.016 (Non-Finnish) 0.17 (Finnish) | Not applicable | no | Likely benign | Targeted  re-seq. |
| 11:118267079 | *UBE4A* | U box | c.3146G>A (NM_004788.2) | Missense | p.Arg1049Gln | Tolerated / Disease caus. | None | rs141017463 | 0.025 (Non-Finnish) | Not applicable | yes  PED.25 | Uncertain sign. | WES-family |
| 11:121340732 | *SORL1* | VPS10p | c.302C>T (NM_003105.5) | Missense | p.Ser101Phe | Deleterious / Disease causing | -1.3% | - | - | 0.26 / 0 | not known | Uncertain sign. | Case-Control |
| 11:121383766 | *SORL1* | VPS10p | c.994C>T (NM_003105.5) | Missense | p.Arg332Trp | Deleterious / Disease causing | None | rs772110877 | 0.010 (Non-Finnish) | 0.26 / 0 | not known | Uncertain sign. | Case-Control |
| 11:121384931 | *SORL1* | VPS10p | c.1112A>C (NM_003105.5) | Missense | p.Asn371Thr | Tolerated / Disease causing | None | rs150609294 | 0.22 (Non-Finnish) 0.24 (Finnish) | 0.26 / 0.58^b^ | no | Likely benign | Case-Control Targeted  re-seq. |
| 11:121391400 | *SORL1* | VPS10p | c.1246C>T (NM_003105.5) | Nonsense | p.Arg416* | Not applicable^a^ | -0.1% | rs144585461 | - | 0.26 / 0 | not known | Likely path. | Case-Control |
| 11:121415993 | *SORL1* | VPS10p | c.1906T>A (NM_003105.5) | Missense | p.Ser636Thr | Deleterious / Disease causing | None | rs138438079 | 0.099 (Non-Finnish) 0.030 (Finnish) | 0.53 / 0.29^c^ | not known | Likely benign | Case-Control |
| 11:121421313 | *SORL1* | VPS10p | c.2200G>A (NM_003105.5) | Missense | p.Asp734Asn | Deleterious / Disease causing | None | rs148430425 | 0.11 (Non-Finnish) 0.87 (Finnish) | 0.53 / 0.29^d^ | not knonw | Likely benign | Case-Control |
| 11:121437647 | *SORL1* | EGF | c.3050-2A<G (NM_003105.5) | Deletion | p.Gly1017-Glu1074del | Not applicable^a^ | -100% | - | - | 0 / 0 | yes  PED.27 | Likely path. | Targeted  re-seq. |
| 11:121458821 | *SORL1* | LDLR class A | c.3907C>T (NM_003105.5) | Missense | p.Arg1303Cys | Deleterious / Disease caus. | None | rs781023219 | 0.0060 (Non-Finnish) | 0 / 0 | yes  PED.25 | Likely path. | WES-family |
| 11:121460051 | *SORL1* | LDLR class A | c.4030T>C (NM_003105.5) | Missense | p.Cys1344Arg | Deleterious / Disease causing | None | - | - | 0.26 / 0 | not known | Uncertain sign. | Case-Control |
| 11:121461856 | *SORL1* | LDLR class A | c.4360C>T (NM_003105.5) | Missense | p.Pro1454Ser | Deleterious / Disease causing | None | rs201304369 | 0.030 (Non-Finnish) 0.33 (Finnish) | 0.53/ 0 | not known | Likely benign | Case-Control |
| 11:121478841 | *SORL1* | Fibronectin type III | c.5195G>C (NM_003105.5) | Missense | p.Gly1732Ala | Deleterious / Disease causing | None | rs777194720 | 0.0075 (Non-Finnish) | 0.26 / 0 | yes  PED.1499 | Uncertain sign. | Case-Control |
| 11:121495911 | *SORL1* | Fibronectin type III | c.6289G>A (NM_003105.5) | Missense | p.Val2097Ile | Deleterious / Disease causing | None | rs74642146 | 0.11 ( Non-Finnish) | 0.26 / 0^e^ | not knonw | Likely benign | Case-Control |
| 12:122018733.. 122018734 | *KDM2B* | not known | c.83_84del (NM_032590.4) | Nonsense | Thr28Serfs*8 | Not applicable^a^ | -4.7% | rs575373073 | 0.42 (Non-Finnish)  0.17 (Finnish) | Not applicable | yes  PED.25 | Likely benign | WES-family |
| ^a^Variant doesn't change the amino acid ^b^In total found in 4 patients and 6 controls ^c^In total found in 3 patients and 2 controls ^d^In total found in 4 patients and 3 controls ^e^In total found in 5 patients and 3 controls | | | | | | | | | | | | | |
