# Supplementary material for: Identification and description of three families with familial Alzheimer disease that segregate variants in the SORL1 gene
Source: Acta Neuropathol Commun. 2017 Jun 9;5:43. doi: 10.1186/s40478-017-0441-9 (PMC5465543; doi:10.1186/s40478-017-0441-9)
Supplement: Supplementary file 4 — Immunohistochemical localization of SORL1 in postmortem brain material. Representative pictures from control, sporadic AD and from two individuals from PED.25 in the CA1 region of hippocampus using four different SORL1 antibodies, AF5699 (a-d) and MAB5699 (e-h), 612633 (i-l) and ab190684 (m-p). Scalebar: 100 μm. Ctrl = control, sAD = sporadic AD, PED.25 II-4= affected family member II:4 and PED.25 II-6= affected family member II:6. (ZIP 36668 kb) [file 40478_2017_441_MOESM4_ESM.zip › 40478_2017_441_MOESM5_ESM_part1.pptx]

## Slide 1
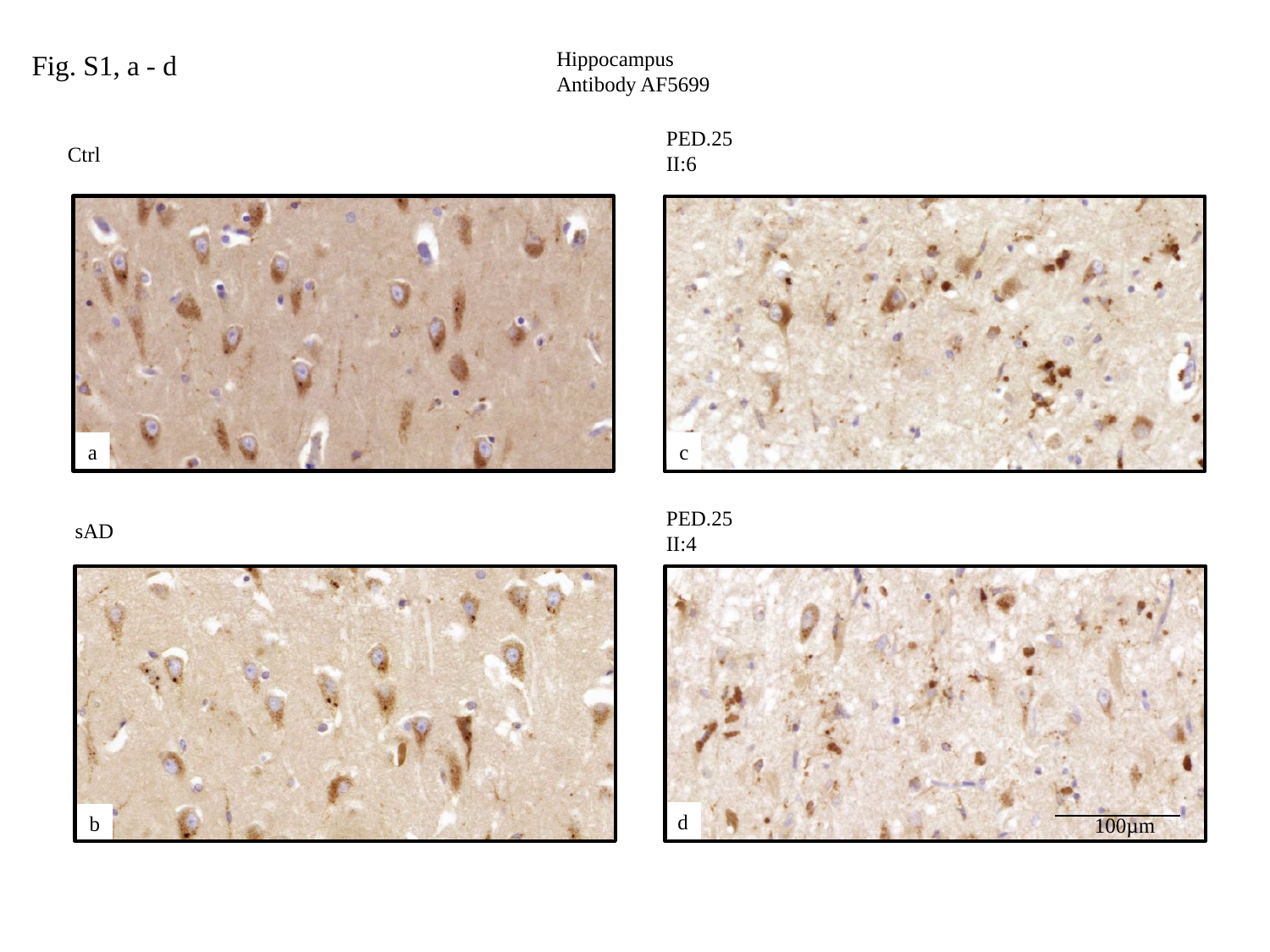

Hippocampus
Antibody AF5699
Fig. S1, a - d
PED.25
II:6
Ctrl
a
c
PED.25
II:4
sAD
d
b
100µm

## Slide 2
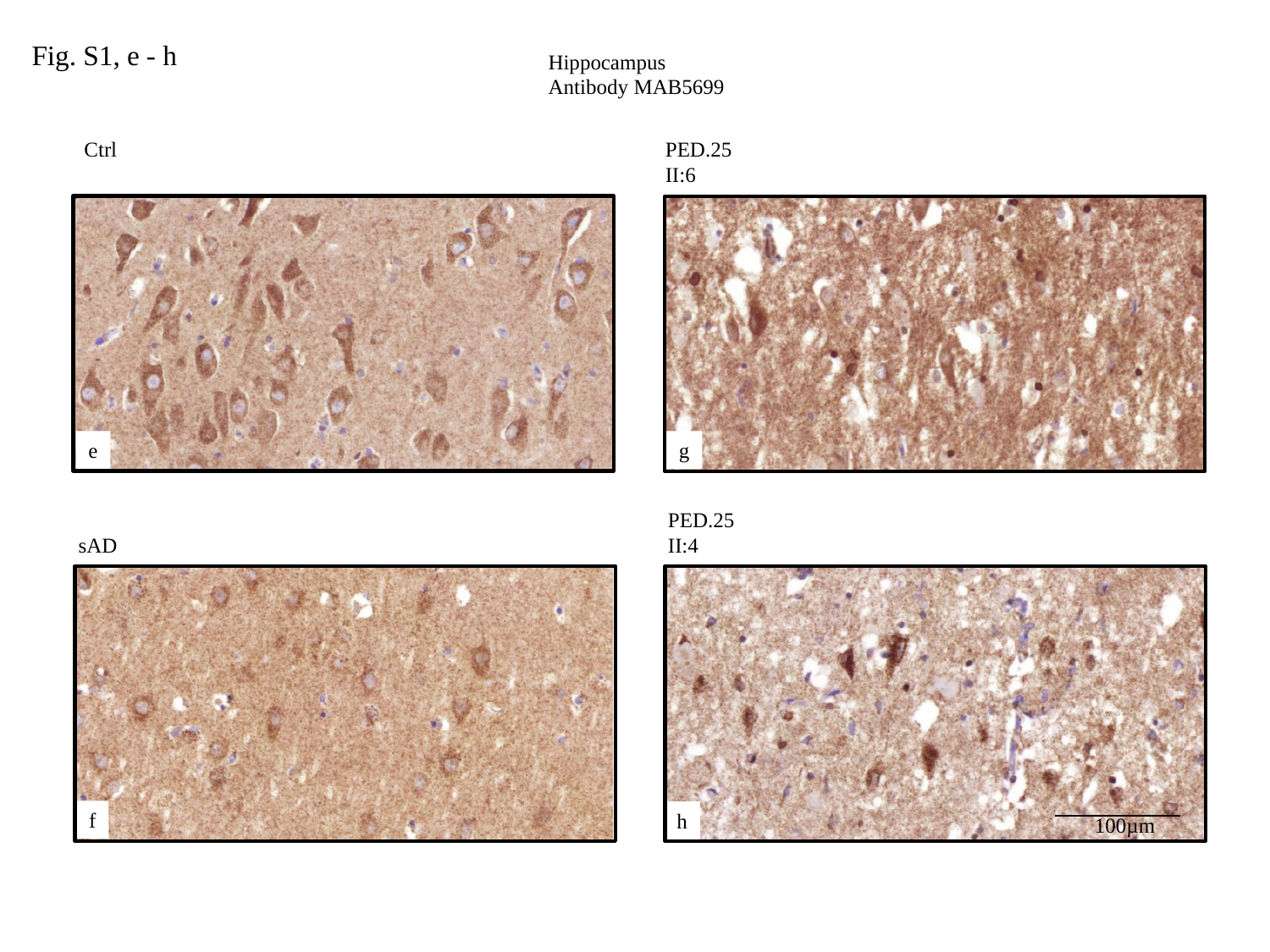

Fig. S1, e - h
Hippocampus
Antibody MAB5699
Ctrl
PED.25
II:6
e
g
PED.25
II:4
sAD
f
h
100µm
